# Selenium enhances chilling stress tolerance in coffee species by modulating nutrient, carbohydrates, and amino acids content

**DOI:** 10.3389/fpls.2022.1000430

**Published:** 2022-09-12

**Authors:** Gustavo F. de Sousa, Maila Adriely Silva, Everton G. de Morais, Gustavo Avelar Z. Van Opbergen, Guilherme Gerrit A. Z. Van Opbergen, Raphael R. de Oliveira, Douglas Amaral, Patrick Brown, Antonio Chalfun-Junior, Luiz Roberto Guimarães Guilherme

**Affiliations:** ^1^Department of Soil Science, Federal University of Lavras, Lavras, Brazil; ^2^Department of Biology, Plant Physiology Sector, Federal University of Lavras, Lavras, Brazil; ^3^Agriculture and Natural Resources, University of California, Hanford, Hanford, CA, United States; ^4^Department of Plant Sciences, College of Agricultural and Environmental Sciences, University of California, Davis, Davis, CA, United States

**Keywords:** environmental changes, beneficial elements, abiotic stress, low temperature, tropical agriculture, plant nutrition, coffee belt

## Abstract

The effects of selenium (Se) on plant metabolism have been reported in several studies triggering plant tolerance to abiotic stresses, yet, the effects of Se on coffee plants under chilling stress are unclear. This study aimed to evaluate the effects of foliar Se application on coffee seedlings submitted to chilling stress and subsequent plant recovery. Two *Coffea* species, *Coffea arabica* cv. Arara, and *Coffea canephora* clone 31, were submitted to foliar application of sodium selenate solution (0.4 mg plant^–1^) or a control foliar solution, then on day 2 plants were submitted to low temperature (10°C day/4°C night) for 2 days. After that, the temperature was restored to optimal (25°C day/20°C night) for 2 days. Leaf samples were collected three times (before, during, and after the chilling stress) to perform analyses. After the chilling stress, visual leaf injury was observed in both species; however, the damage was twofold higher in *C. canephora.* The lower effect of cold on *C. arabica* was correlated to the increase in ascorbate peroxidase and higher content of starch, sucrose, and total soluble sugars compared with *C. canephora*, as well as a reduction in reducing sugars and proline content during the stress and rewarming. Se increased the nitrogen and sulfur content before stress but reduced their content during low temperature. The reduced content of nitrogen and sulfur during stress indicates that they were remobilized to stem and roots. Se supply reduced the damage in *C. canephora* leaves by 24% compared with the control. However, there was no evidence of the Se effects on antioxidant enzymatic pathways or ROS activity during stress as previously reported in the literature. Se increased the content of catalase during the rewarming. Se foliar supply also increased starch, amino acids, and proline, which may have reduced symptom expression in *C. canephora in* response to low temperature. In conclusion, Se foliar application can be used as a strategy to improve coffee tolerance under low-temperature changing nutrient remobilization, carbohydrate metabolism, and catalase activity in response to rewarming stress, but *C. arabica* and *C. canephora* respond differently to chilling stress and Se supply.

## Introduction

Coffee is one of the most important commodities worldwide with a significant economic impact on over 25 million mostly smallholder farmers in more than 60 countries throughout the tropics (Jayakumar et al., 2017). Coffee plants are highly sensitive to the growing environment and are generally restricted to the “Coffee Belt”—between 25 degrees North and 30 degrees South with an average temperature between 18 and 22°C for *Coffea arabica* and 22 and 28°C for *Coffea canephora* (DaMatta and Ramalho, 2006; Descroix and Snoeck, 2004; Bunn et al., 2015; Bliss, 2017). Among the 104 *Coffea* species described (Davis and Rakotonasolo, 2008), the two most economically important species are *C. arabica* (Arabica) and *C. canephora* (Robusta). These two species are responsible for 99% of the world’s green-bean production (Jayakumar et al., 2017).

Changes in the temperature due to climate change might adversely affect coffee plants because each species and genotype requires specific environmental conditions for successful production (Ramalho et al., 2014; Ebisa, 2017). Low-temperature stress may be denominated as (i) cold stress—when plants suffer from sub-zero temperatures, and (ii) chilling stress—when plants suffer from low but non-freezing temperatures (Graves, 1995). As a result of chilling stress, plants have shown reduced stomatal conductance, changes in the pigment complexes and losses of photochemical efficiency, restricted electron transportation, and changes in carbon metabolism, allocation, and partitioning (Ensminger et al., 2006; Partelli et al., 2010; Batista-Santos et al., 2011).

Acclimation to low temperature is usually initiated by a short-term fluctuation in temperature, which affects metabolic homeostasis and induces a stress response (Ensminger et al., 2006). A sudden drop in temperature limits the ability of plants to induce protective metabolic responses. Severe frosts in 2021 were experienced in coffee areas in the southeast of Brazil, the highest production region of Brazil, with almost 8–10% of the arabica coffee affected, which reduced the production in the order of 17% below recent on-year crops (Usda Foreign Agricultural Service, and United States, 2022). Exogenous application of beneficial elements, such as selenium (Se), has emerged as a tool to compensate for the negative impacts of many stresses, including chilling (Brown et al., 2021; Zellner et al., 2021).

Although Se is not an essential element for higher plants, it has been shown to increase antioxidant activity (Ekanayake et al., 2015), change carbohydrate metabolism (Lara et al., 2019; Silva et al., 2020), protect chlorophyll, and modulate water relations (Zhang et al., 2014). Se application has reduced the side effects of abiotic stress in a wide range of staple crops, such as drought in common beans and rice (Andrade et al., 2018; Ravello et al., 2021), heavy metal exposure in wheat (Liu et al., 2021; Hasanuzzaman et al., 2022), and salinity in maize and garlic (Ashraf et al., 2018; Astaneh et al., 2019).

Previous studies resulted in higher coffee yield in response to Se supply by increasing antioxidant metabolism (de Mateus et al., 2021); however, there have been no studies that explore the influence of Se application in coffee species under chilling stress. Here, the effects of Se supply to coffee plants under chilling on plant metabolic responses and plant tolerance were examined.

## Materials and methods

### Plant material

The trial was performed using two different coffee species, *C. arabica* cv. Arara and *C. canephora* clone 31, differing in tolerance to low temperature (DaMatta and Ramalho, 2006). According to these authors, low-temperature tolerance is related to the species’ ability to change its metabolism to trigger adverse conditions (e.g., increases in enzymatic activities, lipids quantitative and qualitative changes, protection of proteins in cell membranes). The plants were provided by the National Institute of Science and Technology of Coffee (INCT *Café*). Plants with 5–6 pairs of fully expanded leaves were used. They were selected for high health and uniformity, and allowed to acclimate under optimal conditions for 14 days in a Conviron^®^ growth chamber [12 h of photoperiod, 60% relative humidity (RH), 260 μmol m^–2^ s^–1^ of light intensity (during the day), and optimal temperature (25°C_*day*_/20°C_*night*_)]. Coffee seedlings were grown on 1 l of a substrate composed of subsoil + cattle manure at a ratio of 3:1, with 5 g of single superphosphate being added to each kilogram of the mixture. The irrigation was made dairy with 80 mL of deionized water during the optimal temperature and 15 mL of deionized water during the chilling temperature.

### Experimental design and treatments

The experiment was arranged in a randomized block design and a 2 × 2 factorial scheme with five replicas of seedlings for each treatment, with the experimental unit consisting of three pots totaling 60 pots. The factorial scheme was composed of two species (*C. arabica* cv. Arara and *C. canephora* clone 31), in the absence and presence of Se (0 and 80 mg L^–1^ Se). Samples were collected three times to evaluate plant responses before, during, and after exposure to chilling stress. Considering the great number of leaves that needed to be collected at each time of evaluation, each replication was composed of three seedlings. The Se rate used in the trial was based on preliminary testing (unpublished data) with coffee seedlings and also on results found for other crops. The control treatment is hereafter described as the plants of the respective species analyzed before being submitted to the chilling stress.

Fourteen days after being transferred to the growth chamber, the plants were moved to a spray chamber to avoid contamination during the foliar treatment application. Thus, the respective Se treatments plants were sprayed manually to drip with 5 mL of a foliar solution of Se (80 mg L^–1^ Se + 0.5% v/v of mineral oil) and the remaining plants were sprayed with mineral oil solution (0.5% v/v of mineral oil). Plants were then returned to the growth chamber. The Se source used was sodium selenate (Na_2_SeO_4_—Sigma Aldrich 98.9%).

The first foliar sampling was performed 7 days after the foliar treatment application. All plants were then exposed to chilling temperatures, which were decreased by 5°C/h from 25 to 10°C during the first day. The temperature was set to 4°C during the night and 10°C during the day (12 h of photoperiod, 60% RH, 260 μmol m^–2^ s^–1^ of light intensity). The temperature regime was defined as suboptimal for coffee growing (Ramalho et al., 2003; DaMatta and Ramalho, 2006).

The second foliar sampling was performed 2 days after low-temperature stress treatment. The temperature was then returned to optimal conditions (25°C_*day*_/20°C_*night*_), and the third sampling was performed 2 days later (post-stress).

### Assessments

#### Visual damage scale

The visual damage from low-temperature exposure in the leaves was carried out according to Manetti Filho and Caramori (1986). The scale of damage ranged from 1 to 5, in this way: (1) no damage; (2) 0–25% of the total leaf area damaged; (3) 25–50% of the total leaf area damaged; (4) 50–75% of the total leaf area damaged; and (5) representing visual damages from 75 to 100% of the total leaf area. The visual damage scale from low-temperature exposure in the leaves was performed considering the general appearance of all leaves.

#### Sample collection and preparation

Two leaf samples were collected for different groups of analyses as follows: (1) The third and fourth fully expanded pairs of leaves from top to bottom of coffee plants were collected and washed three times with distilled water. Then, the samples were dried for 72 h at 60°C and ground in a Willey mill to obtain the dried leaf tissue. The dried samples were used to quantify the parameters described in section 2.3.3 (total content of Se, nitrogen, and sulfur), section 2.3.7 (carbohydrates, total protein, total free amino acids), and section 2.3.8 (proline); and (2) The second fully expanded pair of leaves from top to bottom of coffee leaves were collected 2 h after lights-on then immediately snap-frozen in liquid nitrogen, individually macerated in liquid nitrogen, homogenized in a cooled mortar using 100 mg PVPP (antioxidant), and stored at -80°C. The dried tissue was used to perform the analysis of the content of Se, sulfur, nitrogen, carbohydrates, total protein, total free amino acids, and proline. The frozen tissue was used to quantify the parameters described in sections “Calculation of LOD, LOQ, and reference material recovery” (antioxidant enzymes) and “Antioxidant enzymes (**s**uperoxide dismutase, catalase, **a**scorbate peroxidase, and glutathione reductase)” (hydrogen peroxide and lipid peroxidation).

The sample collection was repeated before, during, and after chilling stress. Since this procedure is a destructive analysis, one plant of the experimental unit was used in each sample collection.

#### Total content of selenium, sulfur, and nitrogen

The extracts for the quantification of Se and S in leaves were obtained by acid digestion of 0.5 g of the dried sample according to the USEPA 3051A protocol (USEPA, 2007) in a microwave (Mars 5, CEM Corporation, Matthews, NC, United States). A blank and certified reference material for Se (White clover, BCR402-IRMM) was included in each batch of samples. The Se content in the leaves was measured using GFAAS (Graphite Furnace Atomic Absorption Spectrometry, Atomic Absorption Spectrometry with Zeeman background correction and EDL lamp for Se; Analyst™ 800 AAS, Perkin Elmer), and the S content was measured using ICP-OES (Inductive Coupled Plasma Emission Spectrometry, Spectro, Blue model, Germany). Total N contents were determined by sulfur digestion and Kjeldahl distillation (Tecnal, TE-136, Brazil) (Malavolta et al., 1997).

#### Calculation of LOD, LOQ, and reference material recovery

The detection and quantification limits (LOD and LOQ) were calculated with three and 10 times the standard deviation (LOD and LOQ, respectively) of 10 individually prepared blank solutions (Silva Junior et al., 2017). The LOD and LOQ for Se were, respectively, 4.26 and 12.2 μg kg^–1^. The Se recovery rate in the reference material was 95.2% ± 4.1.

#### Antioxidant enzymes (superoxide dismutase, catalase, ascorbate peroxidase, and glutathione reductase)

Frozen leaf tissue was weighed (0.2 g) and mixed with 1.5 mL of potassium phosphate buffer solution (0.1 mol L^–1^, pH 7.8 + 0.1 mol L^–1^ EDTA, pH 7.0, 0.01 mol L^–1^ ascorbic acid, and 22 mg polyvinylpolypyrrolidone-PVPP). The suspension was centrifuged at 14,000 *g* for 10 min at 4°C (Biemelt et al., 1998). The supernatant was used to assess the activity of the antioxidant enzymes. Quality assurance and quality control of the enzymatic analyses were warranted by using two blanks in each reading plate and operating the samples at 0–4°C. In addition, the enzyme extraction was performed on the day of the analysis to avoid the oxidation of the enzyme extract.

Superoxide dismutase (SOD, EC 1.15.1.1) activity was evaluated by measuring its ability to inhibit the photochemical reduction of nitro blue tetrazolium at 560 nm (Giannopolitis and Ries, 1977). The reading sample was composed of 50 mM of potassium phosphate buffer, pH 7.8, 14 mM methionine, 0.1 μM EDTA, 75 μM NBT, 2 μL of enzyme extract, and 2 μM riboflavin.

Catalase (CAT, EC:1.11.1.6) activity was assayed by measuring the rate of decomposition of H_2_O_2_ at 240 nm (Havir and McHale, 1987). For this, were pipetted 100 mM of buffer solution of potassium phosphate 200 mM pH 7.0, 12.5 mM H_2_O_2,_ and 3 μL of enzyme extract. The CAT activity was read every 15 s for 3 min and was defined as the amount of enzyme necessary to reduce 1 μmol H_2_O_2_ min^–1^.

Ascorbate peroxidase (APX, EC:1.11.1.11) was determined by the method of reduction of ascorbate at 290 nm (Nakano and Asada, 1981). In order to quantify the APX, 50 mM potassium phosphate 100 mM pH 6.0, 0.8 mM ascorbic acid, 1 mM H_2_O_2_, and 3 μL of the enzyme. The APX activity was read every 15 s for 3 min and was defined as the amount of the enzyme required to oxidize 1 mmol (ascorbate) min^–1^.

Glutathione reductase (GR, EC:1.6.4.2) was assayed according to the methodology proposed by Schaedle and Bassham (1977) and adapted by García-Limones et al. (2002). The GR activity was read at 340 nm. The reaction medium consisted of 50 mM of buffer solution of potassium phosphate pH 7.8, 0.5 mM oxidized glutathione, 3.0 mM MgCl_2_,0.15 mM NADPH, and 15 μL of enzyme extract. One GR unit is defined as the amount of enzyme that oxidizes 1 mmol min^–1^ NADPH.

The analyses were carried out in triplicates and were measured using an Epoch^®^ Microplate Spectrophotometer (BioTek, United States).

#### Hydrogen peroxide and lipid peroxidation (malonaldehyde)

Frozen leaf tissue (0.2 g) was ground in liquid nitrogen, homogenized in 5 mL of trichloroacetic acid (TCA), and centrifuged at 12,000 *g* for 15 min at 4°C. The supernatant was collected to determine hydrogen peroxide (Velikova et al., 2000) with adaptations of Loreto and Velikova (2001). Lipid peroxidation (MDA) was assayed according to Buege and Aust (1978) and Silva et al. (2020).

For the determination of hydrogen peroxide, 0.45 mL of supernatant were added to 2.5 mM potassium phosphate buffer, pH 7.0, and 0.5 mM potassium iodate. The absorbance of the supernatant was read at 390 nm. The content of H_2_O_2_ was calculated by comparison with a standard calibration curve previously made using different concentrations of H_2_O_2_.

The assay of lipid peroxidation (MDA) was carried out by the thiobarbituric acid (TBA) test, which determines the MDA as an end product of lipid peroxidation. Then, 0.125 mL of the supernatant was added to 0.25 mL of a mixed solution of TBA (0.5%) and TCA (10%). The mixture was incubated in a water bath at 95°C for 30 min, and the reaction was stopped by placing the reaction tubes in an ice bath. The absorbance of the supernatant was measured at 532 nm, subtracting the value for non-specific absorption at 600 nm. This procedure was made in duplicates.

#### Carbohydrates, total protein, and total free amino acids

The extraction of carbohydrates and proteins was based on Zanandrea et al. (2010). Individual dried leaf samples were weighed (0.2 g), mixed with 5 mL of potassium phosphate buffer (pH 7.0), and heated in a water bath at 30°C for 40 min. Then, the suspension was centrifuged at 10,000 *g* for 20 min and the supernatant was collected. This procedure was done twice and both supernatants were mixed. The same pellet was used for starch extraction mixing 8 mL of potassium acetate buffer (200 mM pH 4.8) and 2 mL of amyloglucosidase (1 mg mL^–1^; 16 units of enzyme). Then, the samples were heated in a water bath at 40°C for 120 min and centrifuged for 20 min at 10,000 *g*. The supernatants were collected for measurements. The contents of starch, sucrose (Suc), and total soluble sugars (TSS) were determined using the anthrone method (Dische, 1962). Reducing sugars were determined according to the DNS method (Miller, 1959), and total free amino acids (AA) were determined according to the ninhydrin method (Yemm et al., 1955). The protein content (Prt) in the leaves was also determined (Bradford, 1976).

#### Proline

Proline content was assessed by the colorimetric method originally described by Bates et al. (1973) with minor modifications. The dried leaf tissue (0.1 g) was weighed and macerated with sulfosalicylic acid 3%. Next, samples were mixed for 60 min at environmental temperature. After the extraction, the content of Pro in the leaves was determined by adding 0.5 mL of extract, 1.5 mL of deionized water, 2 mL of a freshly prepared acid-ninhydrin solution, and 2 mL of pure acetic acid. Tubes were incubated in a water bath at 100°C for 60 min. The reaction was stopped by placing the reaction tubes in an ice bath. The supernatant was carefully collected and read at 520 nm.

### Statistical analysis

The statistical analyses were performed using the R software (R Core Team, 2021). An exploratory analysis of data was first performed to verify the existence of outliers. Then, the analysis of variance (ANOVA) was conducted on the data after the validation of the model and tests of assumptions (normality, homoscedasticity, independence, and additivity of residuals). When significative (*p <* 0.05), the interaction of the studied factors (Se supply and coffee genotypes) was compared. When there was no interaction between tested factors (*p >* 0.05), the means of the treatments were compared at each factor. Means were compared using the Tukey test (*p* < 0.05). In addition, principal component analyses (PCAs) were performed to determine the relationships of the measured variables. Pearson’s correlation analysis (*p* < 0.05) was performed to validate the relationships observed in PCA. PCA and correlation analysis was performed for each species and time of evaluation (before, during, and after stress). The correlation matrices among variables are reported in Supplementary Material.

## Results

### Visual damage scale

**FIGURE 1:**
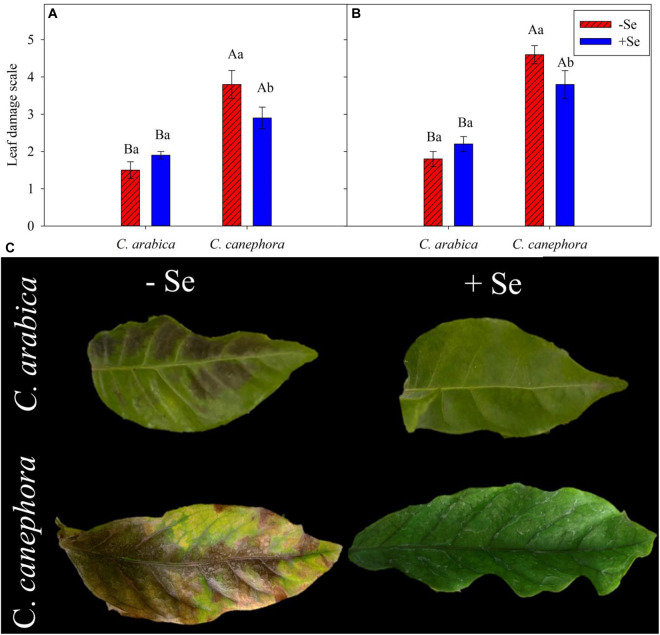
Leaf visual damage in coffee species exposed to chilling stress and two conditions of Se foliar supply after 2 days of exposure to low temperature. Visual damage scale during **(A)** and after stress **(B)** according to Manetti Filho and Caramori (1986). **(C)** Visual damage in coffee after low-temperature stress. Mean values followed by different lowercase letters within Se supply conditions (with selenium and without Se) in each genotype and different uppercase letters indicate significant differences within each genotype (*C. arabica* and *C. canephora*) in each Se supply condition are significantly different (*p <* 0.05, *n* = 5) by Tukey multiple comparison test. Vertical bars represent the standard error.

Leaf visual damage was influenced by species and Se supply (Figure 1). *C. canephora* was statistically (*p* < 0.05) more affected than *C. arabica* at both evaluation times. During the stress, the damage to *C. canephora* was twofold higher than in *C. arabica* (Figure 1A). Se supply reduced the damage by low temperature in *C. canephora* by 24 and 17% compared with its initial control value at optimal temperature (25°C day/20°C night), respectively, for the evaluations performed during chilling and the rewarming (Figures 1A,B).

*C. canephora* showed main leaf damage in the leaves with a yellowish-green color during and after the cold stress (Figure 1C). Although the *C. arabica* did not show high damage by cold, slight darkened damage in the leaves after 2 days of exposure to chilling stress was observed (Figure 1C).

### Analysis of selenium, sulfur, and nitrogen

**FIGURE 2:**
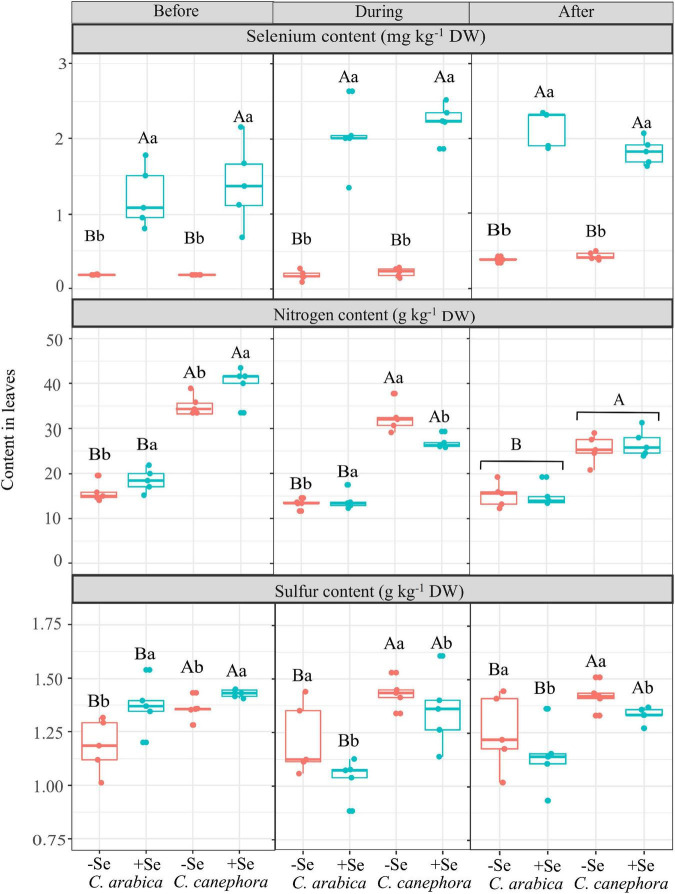
Effects of Se foliar application and temperature condition on Se, S, and N content in leaves of *C. arabica* and *C. canephora*. Mean values followed by different lowercase letters within Se supply conditions (with selenium and without Se) in each genotype are significantly different (*p <* 0.05, *n* = 5) by Tukey multiple comparison tests as well as different uppercase letters that indicate significant differences in species (*C. arabica* and *C. canephora*). Vertical bars represent the standard error.

Leaf Se content ranged from 0.18 mg kg^–1^ DW (control treatment) to 2.13 mg kg^–1^ DW (after chilling stress) in the *C. arabica* and 0.18 mg kg^–1^ DW (control treatment) to 1.81 mg kg^–1^ DW (after chilling stress) in the *C. canephora*. There was no statistical difference between the species (Figure 2).

In this study, Se foliar supply increased the N content in the leaves before plants were submitted to chilling stress, but N content was reduced in the low-temperature condition by Se application (Figure 2).

The leaf S content was affected by species and Se supply in all the evaluation times (*p* < 0.05). The S content in *C. canephora* was significantly higher than in *C. arabica*. Se foliar supply promoted 9% higher S content in leaves on the evaluation performed before the cold, but Se supply reduced the S content in the leaves during and after stress (Figure 2). The S content decreased 10.5 and 10.7%, respectively, during and after chilling stress by Se application.

### Antioxidant enzymes (superoxide dismutase, catalase, ascorbate peroxidase, and glutathione reductase)

**FIGURE 3:**
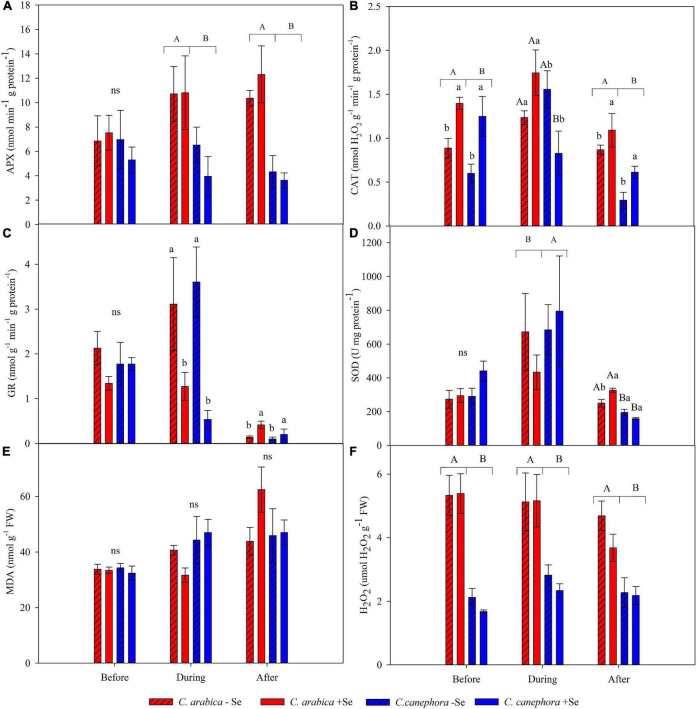
Effects of Se foliar application on APX **(A)**, CAT **(B)**, GR **(C)**, SOD **(D)**, MDA **(E)**, and H_2_O_2_ **(F)** in leaves of *C. arabica* and *C. canephora* during the rewarming. Mean values followed by different lowercase letters within Se supply conditions (with selenium and without Se) in each genotype are significantly different (*p <* 0.05, *n* = 5) by Tukey multiple comparison tests as well as different uppercase letters that indicate significant differences in species (*C. arabica* and *C. canephora*). Vertical bars represent the standard error.

The average values of antioxidant enzyme activity (GR, SOD, CAT, and APX), as well as the hydrogen peroxide (H_2_O_2_) and lipid peroxidation (MDA), assessed in the species treated and non-treated with foliar Se are presented in Figure 3 and Supplementary Table 1.

Chilling stress promoted an increase of 44% in GR activity in non-treated plants with Se, but these plants were unable to keep high GR activity during the rewarming condition and the GR activity was reduced by 97% (Figure 3C and Supplementary Table 1). On the other hand, the Se supply was responsible for statically increasing the GR after the chilling stress compared with non-treated plants with Se.

The SOD activity was notably increased during chilling stress compared with optimal temperature conditions. After stress, SOD was affected by the interaction of the two factors (Species × Se supply). Foliar supply promoted 23.5% higher SOD activity in *C. arabica* (Figure 3D and Supplementary Table 1). The same effect was not shown in the *C. canephora.*

Foliar supply of Se promoted 50% less CAT activity in *C. canephora* than the same non-treated species during chilling stress. Moreover, Se foliar supply increased CAT activity before and after the stress, independently of the species (*p <* 0.05). APX activity was not influenced by Se application and was affected by the species in which *C. arabica* showed higher activity regardless during and after chilling (Figure 3A and Supplementary Table 1).

### Hydrogen peroxide and lipid peroxidation (malonaldehyde)

Levels of malonaldehyde (MDA) and H_2_O_2_ were not influenced by the presence of Se and species (Figures 3E,F and Supplementary Table 1). The stress increased the MDA content by 7.7 and 35.6% in the *C. arabica* and *C. canephora* compared with the respective genotype before the stress. After the stress, MDA increased by 58.3 and 38%, respectively, for *C. arabica* and *C. canephora* compared with the same species before stress. This supports the hypothesis that *C. canephora* has less ability to tolerate low temperatures than *C. arabica* because the MDA content increased promptly after the plants were submitted to chilling stress. On the other hand, MDA content in the *C. arabica* showed subtle adjustment during the stress but increased abruptly from 36.2 to 53.2 nmol g^–1^ FW^–1^ during the rewarming.

### Carbohydrates, total protein, and amino acids

The Suc content in leaves was affected by the species in all periods of evaluation and *C. arabica* had higher Suc content than *C. canephora.* In addition, *C. arabica* showed less impact from chilling stress on Suc (Supplementary Table 2).

*C. canephora* showed less ability to maintain the initial content of Suc and RS after exposure to low temperature than *C. arabica*. The reduction of Suc and RS in the *C. canephora* was 22.7 and 25.7%, respectively. During rewarming, the *C. canephora* plants were unable to increase the Suc and RS content as *C. arabica*, showing a reduction of 45.2 and 44.2% compared with the plants before the stress. The *C. arabica* plants also showed a subtle reduction in Suc when exposed to chilling stress, but it was less pronounced than in *C. canephora*. Meanwhile, the *C. arabica* plants reduced the RS content in the leaves during the stress, but its content was increased by 8.8% in the rewarming period.

**FIGURE 4:**
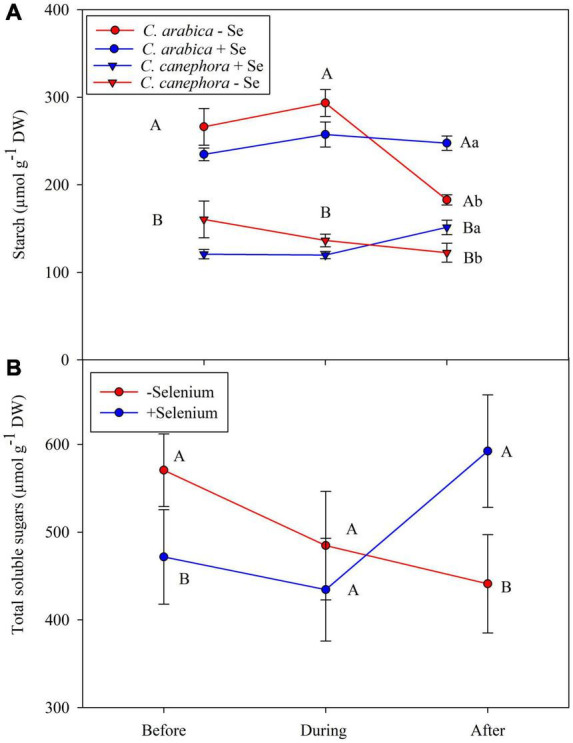
Effects of Se foliar application and temperature conditions on Starch **(A)** and TSS **(B)** content in leaves of *C. arabica* and *C. canephora*. The TSS content was obtained by the average of both species. Mean values followed by different lowercase letters within Se supply conditions (with selenium and without Se) in each genotype are significantly different (*p <* 0.05, *n* = 5) by Tukey multiple comparison tests as well as different uppercase letters that indicate significant differences in species (*C. arabica* and *C. canephora*). Vertical bars represent the standard error.

The Se foliar application promoted lower starch content in the plants before and during stress, but its supply modulated the starch content after the plants were subjected to chilling stress, which led to an increase of ∼30.7% in the starch when compared with plants that did not receive Se foliar application (Figure 4A). In addition, Pearson’s correlation analysis showed a positive correlation (*p <* 0.05) of Se and starch in both species after the chilling stress—*R*^2^ = 0.92 and *R*^2^ = 0.68, respectively, to *C. arabica* and *C. canephora* (Supplementary Figures 5, 6). There were significant differences (*p <* 0.05) between Se supply in the TSS content before and after the stress. The TSS content in foliar tissue from Se-supplied plants was 18% lower than in those that did not receive Se supply (Figure 4). Foliar supply reduced the TSS content before stress. In contrast, the Se supply increased the TSS content after the chilling stress in both species. After the stress, TSS showed a correlation with Se content in the leaves according to PCAs (Figures 4E,F). This behavior is also supported by a significant correlation (*p <* 0.05) to Se content in leaves in both species according to Pearson’s correlation analysis (Supplementary Figures 5, 6).

The application of Se improved the Prt content in *C. arabica* leaves before the plants were submitted to chilling stress, but this effect was not noticed during the chilling stress and the rewarming period. Despite this, Prt was higher in *C. canephora* than in *C. arabica* during all growth temperature conditions. Similarly, the AA content was higher in *C. canephora* than in *C. arabica*, where the AA content was not influenced by Se application before and after the chilling stress.

### Proline

**FIGURE 5:**
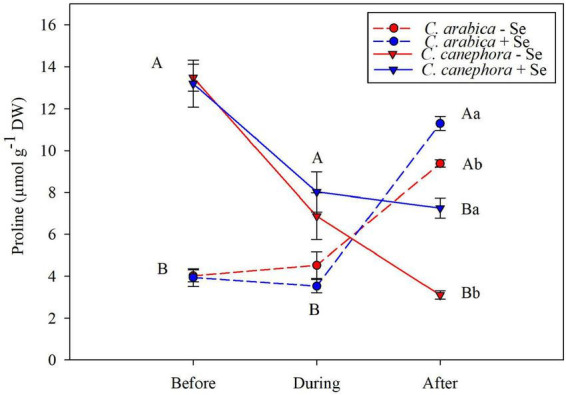
Effects of Se foliar application and temperature conditions on Pro content in leaves of *C. arabica* and *C. canephora* during the rewarming. Mean values followed by different lowercase letters within Se supply conditions (with selenium and without Se) in each genotype are significantly different (*p <* 0.05, *n* = 5) by Tukey multiple comparison tests as well as different uppercase letters that indicate significant differences in species (*C. arabica* and *C. canephora*). Vertical bars represent the standard error.

The Pro content was affected by species before and during chilling stress, in which *C. canephora* has shown notably higher content than *C. arabica*. Nevertheless, Pro content in *C. canephora* during the stress was reduced by 44 % after the stress, showing that the low temperature can exert great influence on the Pro content in stress conditions. Despite the lower initial Pro content in the *C. arabica*, this genotype was able to increase significantly the content in the rewarming, which was potentialized by the Se application. Se application increased 20.4 and 133% of the Pro content, respectively, to *C. arabica* and *C. canephora* without Se application (Figure 5).

### Principal component analysis

**FIGURE 6:**
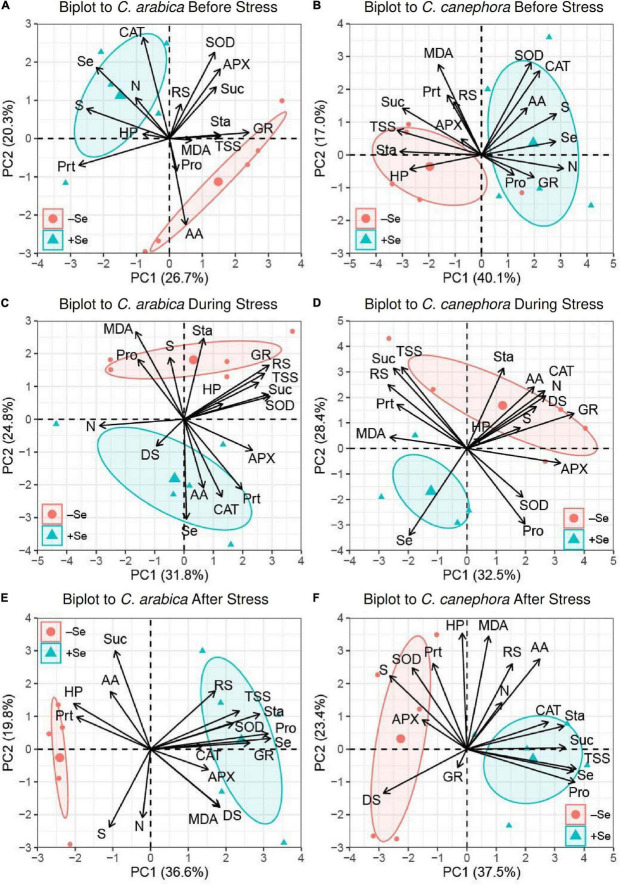
PCAs biplot representation of leaves composition data before, during, and after chilling stress in two species. **(A,C,E)** *C. arabica* before, during, and after stress, respectively; **(B,D,F)** *C. canephora* before, during, and after stress, respectively.

The principal component analysis (PCA) showed that the relations between the analyzed parameters and Se content in leaves vary as a function of species and temperature conditions (before, during, and after chilling stress). Overall, it is possible to find two groups enclosed in the ellipses, which are composed of samples supplied with Se at all evaluation times (Figure 6).

Before stress in *C. arabica*, the application of Se promoted the higher contents of Se, and this Se had a positive relationship with CAT and the content of S and a negative relation with AA (Figure 6). During stress, the positive relationship between Se content and CAT was maintained, with CAT having also a positive correlation with Prt. After chilling stress, the relationship between CAT and Se content was not maintained. Se content was increased by its application and had a positive correlation with Pro, TSS, Sta, GR, and SOD.

For *C. canephora* after chilling, the PCA showed a positive and significant correlation (*p <* 0.05) of Se with CAT, Sta, Suc, TSS, and Pro, which was supported by the correlation matrix (Supplementary Figure 6).

A negative relationship was observed between S and TSS content. During the low-temperature stress, the Se content showed a negative relationship with the content of N, AA, Sta, CAT, and GR. After the stress in *C. canephora*, Se content had a positive relationship with Pro, TSS, Sta, Suc, and CAT.

## Discussion

The stress promoted by chilling impacted negatively plant development and caused significant damage to the leaves. Plant exposure to low-temperature stress commonly reduces the physiological parameters (e.g., stomatal conductance, photosynthetic rate, and intercellular CO_2_ concentration) (Huang et al., 2018). Stomatal closure has been related as one of the first plant mechanism responses to cold stress, conferring stress tolerance during low-temperature stress and the reduction of CO_2_. Low CO_2_ fixation can reduce the photosynthetic rate by causing disequilibrium between light capturing and utilization, as well as by changing the photochemistry of chloroplasts. The excess light energy in the photosystems causes an imbalance between electron release and acceptance, which increases ROS formation (Larcher, 1985; de Oliveira et al., 2002).

Partelli et al. (2009) showed that increased ROS promotes lipidic peroxidation and loss of membrane selectivity, then, coffee plants submitted to low temperature have shown chlorophyll loss and leaf tissue degradation, reflecting injuries in the leaves. These leaf damages were also observed in this trial (Figure 1). According to Huang et al. (2018), the foliar supply of Se reduced significantly the foliar damage of chilling stress in strawberries, and the authors conferred this behavior to the enhanced gas exchange during the stress. Their results also showed that Se alleviated chlorophyll degradation and reduced MDA and H_2_O_2_. Additionally, it has been shown that Se application increases the stability of the photosynthetic machinery, while also preserving the membrane system, which promotes higher tolerance to low temperatures (Yang et al., 2021).

The higher damage in the *C. canephora* leaves compared with *C. arabica* suggests that each genotype might act distinctly when submitted to stress in the triggering of metabolic responses to temperature changes (Petek et al., 2005; Fortunato et al., 2010; DaMatta et al., 2018), including different responses to Se application. These results are explained by the allopolyploidy of *C. arabica*, which promotes an evolutionary advantage in having additional genetic materials that attribute greater plasticity in coping with environmental variations compared with its parentals—in this case, *C. canephora* and *C. eugenioides*. In other words, the allopolyploidy of *C. arabica* makes this species able to up and downregulate certain genes responsible to keep the homeostasis during low temperatures, as reported by Bardil et al. (2011), or even at higher temperatures (de Oliveira et al., 2020).

The increase in the S content in the coffee leaves before the chilling stress probably occurred due to its intimate relation with Se metabolism in plants. Currently, some studies have shown that the high-affinity sulfate transporters involved in sulfate uptake and translocation throughout plant tissues may be utilized by selenate (NaSeO_4_) as well (Sors et al., 2005; White, 2018). At this point, low content of Se can improve S uptake by mimicking S deficiency to activate specific sulfate transporter expression and stimulate S uptake, resulting in the selenate-induced S accumulation (Boldrin et al., 2016).

The higher S content in leaves led to an increase in the Prt before stress, which was supported by significative Pearson’s correlation (*R*^2^ = 0.80, *p <* 0.05) in the *C. arabica* (Supplementary Figure 1). Sulfur is a structural constituent of certain amino acids (e.g., methionine; Met and cysteine; Cys) and coenzymes, as well as in prosthetic groups such as ferredoxin, essential composts for plants to survive in unfavorable conditions (Saleem et al., 2021).

In addition, S composes the amylase molecule through Cys. Since Cys compose the amylase, this amino acid can increase amylase activity aiming to face the stress. Then, most of the stored source of carbohydrates is degraded by amylase and the product is then supplied to the plants for energy and carbon for growth (Thalmann and Santelia, 2017). Meanwhile, Se supply can also stimulate amylase by the same mechanisms endorsed by S, since they share the same primary metabolism in plants and Se can be incorporated in Cys, giving rise to Se-Cys (Jacob et al., 2003; White, 2018).

The reduction of S content during and after chilling stress by Se supply could be connected with the potential changes in energetic metabolism of plants under severe stress, which cause its remobilization from leaves to storage parts, such as roots and stems. The storage of nutrients may be an effective alternative for sustaining plant growth and plays a key role in energy-saving during the rewarming condition (Etienne et al., 2018).

Lipid peroxidation is a good indicator of ROS activity on cell damage, mainly because oxidative stress causes the peroxidation of unsaturated fatty acids, whereas increasing MDA concentration (Farooq et al., 2019). Exogenous Se supply has also been related to reduced ROS, such as H_2_O_2_, and lipidic peroxidation under stress conditions (Jóźwiak and Politycka, 2019; Silva et al., 2020; de Mateus et al., 2021). However, this behavior was not seen in this trial with coffee (Supplementary Table 1). Despite this, the Se supply significantly reduced injuries to the leaf tissue of *C. canephora* plants during and after the stress (Figure 1), suggesting that the negative effects of chilling stress are mitigated by pathways other than ROS scavenging.

The effect of Se on the improvement of antioxidant enzyme activity during chilling stress has been reported elsewhere (Chu et al., 2010; Abbas, 2012). In fact, plants can acclimate—i.e., can adjust to changes in their environment— to some extent, sustaining effective metabolism as a result of a variety of complimentary mechanisms that defend the cell. However, under extreme conditions plants may not be able to adapt to environmental disturbances, which results in severe damage to cell structures and also to proteins involved in the physiological metabolism (DaMatta et al., 2018). In addition, antioxidant enzymes are highly dependent on protein functions and low temperatures can lead the proteins to reduce their activity and lower cellular fluidity (Maksimov et al., 2017).

The higher content of Se in leaves and the remobilization of S from shoot to roots probably are correlated with de TSS, starch, AA, and Pro content during the rewarming (Supplementary Table 2 and Figure 4). It can be suggested based on data that to keep the carbohydrate demand for growth under low temperature, Se can help plants to remobilize the S from leaves after the stress.

In this way, Se application helped the plants to maintain the starch content during the rewarming, since Se increased starch content by 12% compared with the same treatment before the chilling stress (Figure 4 and Supplementary Table 2). On the other hand, control treatments showed a reduced starch content by 28%. These results show that foliar Se cannot only reduce the starch breakdown but also increase the content after the low-temperature stress compared with those that do not receive foliar Se.

Provided that the effect of chilling stress includes impairment of photosynthesis, Se supply in plants cause increases in the structure and functionality of the photosynthetic apparatus, allowing the plants to maintain higher net photosynthesis during stress condition (Lara et al., 2019; Souza et al., 2019). At this point, transitory starch is synthesized in the leaves directly from photosynthates during the day and can be degraded the following night to sustain metabolism, energy production, and biosynthesis in the absence of photosynthesis (Pfister and Zeeman, 2016). According to Stein and Granot (2019) and Ribeiro et al. (2022), starch not only acts in the energetic metabolism, but also as promoting rapid stomatal opening, making osmoprotectants, cryoprotectants, scavengers of free radicals and signals, and reverting embolized vessels. Besides, its cleavage products are available for many metabolic pathways, including the synthesis of complex carbohydrates.

According to PC1, during the rewarming, the effect of Se on Suc was positive in *C. canephora*, but negative in *C. arabica*. Moreover, Suc was found on the opposite side of DS in PCA1 (Figure 6F) and also significantly negative according to Pearson’s correlation (*R*^2^ > 0.74) (Supplementary Figure 6). In addition to higher Suc, the Se application also promoted higher TSS, total amino acids (AA), and Pro content in leaves, regardless of genotype during the rewarming (Supplementary Table 2). These results evidence that, although *C. canephora* plants were not able to maintain their full development during the stress, Se supply can impair plant metabolism after the low-temperature stress, which results in less damage to *C. canephora* plants.

Proline content was affected by the species before and during low-temperature stress and *C. canephora* showed higher content than *C. arabica*. Nevertheless, the *C. canephora* reduced the content of Pro by 43% when submitted to low temperature, and 60% during the rewarming. Meanwhile, the Pro content in *C. arabica* maintained the same status during chilling stress but increased by 15% compared with Pro content before stress. Although Se affected positively the Pro content in both species, it is remarkably in *C. canephora* (135%) when compared with *C. arabica* (20%).

The considerable depletion of Pro content in *C. canephora* showed that this specie had less ability to survive during the stress. In contrast, *C. arabica* was able to modulate the content of Pro to protect the cellular structures and reduce the production of ROS. It is also supported by the allopolyploidy of *C. arabica*, in which these plants are able to activate different genes to induce the production of Pro in the rewarming and downregulate its content in the *C. canephora*. Moreover, the regulation of these genes can also be dependent on the external stimulus, which was remarkably changed by temperature and/or Se supply (Krishnan et al., 2008; Ni et al., 2009; Bardil et al., 2011).

As a result of chilling stress, the plants are submitted to osmotic constrictions due to the reduced uptake of water. Then, the soil water potential progressively decreases, hampering and eventually halting the gradient of water flow from roots to apical shoot. The resulting osmotic stress may cause stomatal closure, reduced photosynthesis rate, growth inhibition, and ROS accumulation (Trovato et al., 2008). A response to osmotic stress widespread in plants consists in the accumulation of compatible osmolytes, such as Pro, which are thought to protect cells against stress damage.

The catabolism of Pro occurs in the mitochondria and it is connected to oxidative respiration and administers energy to resume growth after stress. During energy depletion, Pro might be oxidated to glutamate by flavin-dependent proline-dehydrogenase (PRODH) and NAD^+^-dependent P5C dehydrogenase (P5CDH), which are two enzymes found in the mitochondria (Liang et al., 2013; Qamar et al., 2015; Zhang and Becker, 2015). Thus, the oxidation of Pro contributes to mitochondrial metabolism and ATP production by providing carbon skeletons and saving extreme energy depletion (Hildebrandt et al., 2015). The Pro behavior in this trial is supported by its negative correlation with TSS, and Suc during the stress with *C. canephora*, which showed *R*^2^ = −0.77 and *R*^2^ = −0.79, respectively (*p* < 0.05) (Supplementary Figure 4). In this case, the *C. canephora* reduced the Pro content during the stress to maintain the carbohydrates contents as an energetic source, avoiding carbohydrate starvation.

The PCA showed that Se supply responses vary not only in the species but also in different temperature conditions. It is also important to highlight that none of the analyzed variables showed a positive correlation with Se content in leaves during stress with *C. canephora* plants according to PCA1 (28.4%) and PCA2 (32.5%) (Figure 4D). Figure 4D shows that the variables analyzed presented a neutral or negative correlation with Se content. The absence of positive correlation during the stress is probably due to metabolic dysfunctions in *C. canephora* during low temperature, which resulted in higher injuries in the leaves.

Plant cells can sense chilling stress through low-temperature-induced changes in membrane fluidity, protein, nucleic acid conformation, and/or metabolite concentration (a specific metabolite or redox status) (Chinnusamy et al., 2007). Low temperature can inhibit the activities of some antioxidant enzymes (e.g., GR) that protect plants against ROS. The reduction of GR during the low temperature was not observed in the treatment of *C. canephora* without Se application. In this treatment, the GR increased 78% during the chilling stress compared with the same treatment before the stress (Supplementary Table 1). However, after the chilling stress, the Se application promoted three times more GR activity in plants when compared with those that did not receive Se. In other words, plants without Se were unable to maintain the GR activity after chilling stress.

## Conclusion

Our findings showed a considerable depletion of plant metabolism at low temperature in both of the species studied, resulting in leaf damage and lipidic peroxidation (MDA), notably higher in *C. canephora*. The cold makes plants unable to trigger metabolic responses during the stress, reducing the content of carbohydrates and AA. Despite this, foliar Se application improved plants’ odds of survival and reduced the leaf’s injuries largely through enhancement in increasing the content of carbohydrates (TSS, starch, and Suc) and AA in the rewarming. All these compounds might also work as cryoprotective substances toward cold-sensitive enzymes, avoiding high membrane rigidity and also maintaining the membrane structure. Therefore, the application of Se at lower levels could be suggested as an important strategy for improving coffee development during cold, helping the plants to recover from the low-temperature stress. New trials focused on the impact of Se on gene expression and associated thermotolerance should be conducted to elucidate the role of this beneficial element on plant metabolism aiming at clarifying these results.

## Data availability statement

The original contributions presented in this study are included in the article/Supplementary material, further inquiries can be directed to the corresponding author/s.

## Author contributions

GS, MS, RO, AC-J, and LG designed the research. GS, MS, GZV, and GAV conducted the experiments and chemical analyses. GS, MS, and EM analyzed the data. GS and MS wrote the original draft. RO, PB, DA, AC-J, and LG wrote the final text and approved the final version of the manuscript. All authors contributed to the article and approved the submitted version.
