# Supplementary material for: Selenium enhances chilling stress tolerance in coffee species by modulating nutrient, carbohydrates, and amino acids content
Source: Front Plant Sci. 2022 Sep 12;13:1000430. doi: 10.3389/fpls.2022.1000430 (PMC9511033; doi:10.3389/fpls.2022.1000430)

Supplementary Material

Table 1. Effects of Se and temperature conditions on superoxide dismutase (SOD, EC 1.15.1.1), catalase (CAT, EC:1.11.1.6), ascorbate peroxidase (APX, EC:1.11.1.11), glutathione reductase (GR, EC:1.6.4.2), lipidic peroxidation (MDA), and hydrogen peroxide (H_2_O_2_). Mean values are significantly different (*p<*0.05, n=5) by Tukey multiple comparison test.

| GR (nmol g^-1^ min^-1^ g protein^-1^) | | | | | | | | | |
| --- | --- | --- | --- | --- | --- | --- | --- | --- | --- |
|  | Before | | | During | | | After | | |
|  | -Se | +Se | Mean | -Se | +Se | Mean | -Se | +Se | Mean |
| *C. arabica* | 2.1ns | 1.3ns | 1.7ns | 3.1ns | 1.3ns | 2.2ns | 0.1ns | 0.4ns | 0.3ns |
| *C. canephora* | 1.8ns | 1.8ns | 1.8ns | 3.6ns | 0.5ns | 2.1ns | 0.1ns | 0.2ns | 0.2ns |
| Mean | 1.9ns | 1.56ns |  | 3.4a | 0.9b |  | 0.1b | 0.3a |  |
| SOD (U mg protein^-1^) | | | | | | | | | |
| *C. arabica* | 273.3ns | 295.0ns | 284.1ns | 672.4ns | 433.2ns | 552.8A | 249.8Ab | 326.4Aa | 288.1A |
| *C. canephora* | 289.9ns | 440.8ns | 365.3ns | 684.2ns | 794.6ns | 739.4B | 195.2Ba | 158.6Ba | 176.9B |
| Mean | 281.6ns | 367.9ns |  | 678.3ns | 613.9ns |  | 222.5ns | 242.5ns |  |
| CAT (nmol H_2_O_2_ g^-1^ MF min^-1^ g protein^-1^) | | | | | | | | | |
| *C. arabica* | 0.9ns | 1.4ns | 1.1A | 1.2Aa | 1.7Aa | 1.5ns | 0.9ns | 1.1ns | 1.0A |
| *C. canephora* | 0.6ns | 1.3ns | 0.9B | 1.6Aa | 0.8Bb | 1.2ns | 0.3ns | 0.6ns | 0.5B |
| Mean | 0.9b | 1.3a |  | 1.4ns | 1.3ns |  | 0.6b | 0.9a |  |
| APX (nmol AsA min^-1^ g protein^-1^) | | | | | | | | | |
| *C. arabica* | 6.9ns | 7.5ns | 7.2ns | 10.7ns | 10.8ns | 10.8A | 10.4ns | 12.3ns | 11.3A |
| *C. canephora* | 7.0ns | 5.3ns | 6.1ns | 6.5ns | 4.0ns | 5.2B | 4.3ns | 3.6ns | 4.0B |
| Mean | 6.9ns | 6.4ns |  | 8.6ns | 7.4ns |  | 7.4ns | 8.0ns |  |
| MDA (nmol g FW^-1^) | | | | | | | | | |
| *C. arabica* | 33.8ns | 33.5ns | 33.6ns | 40.6ns | 31.7ns | 36.2ns | 43.9ns | 62.5ns | 53.2ns |
| *C. canephora* | 34.3ns | 32.4ns | 33.7ns | 44.3ns | 47.0ns | 45.7ns | 45.9ns | 47.0ns | 46.5ns |
| Mean | 34.1ns | 32.9ns |  | 42.5ns | 39.3ns |  | 44.9ns | 54.8ns |  |
| H_2_O_2_ (µmol H_2_O_2_ g FW^-1^) | | | | | | | | | |
| *C. arabica* | 5.3ns | 5.4ns | 5.4A | 5.1ns | 5.2ns | 5.1A | 4.7ns | 3.7ns | 4.2A |
| *C. canephora* | 2.1ns | 1.7ns | 1.9B | 2.8ns | 2.3ns | 2.6B | 2.3ns | 2.2ns | 2.2B |
| Mean | 3.7ns | 3.5ns |  | 4.0ns | 3.8ns |  | 3.5ns | 3.0ns |  |

Mean values followed by different lowercase letters within Se supply conditions (-Se or +Se) in each genotype are significantly different (*p<*0.05, n=5) by Tukey multiple comparison test as well as different uppercase letters that indicate significant differences in genotypes (*C. arabica* and *C. canephora* ). ns = non-significance by Tukey multiple comparison test (*p<*0.05, n=5).

Supplementary Table 2. Effects of Se and temperature conditions on sucrose, reducing sugars (RS), total soluble sugars (TSS), starch, protein, and total amino acids (AA).

| Sucrose (µmol of glucose g^-1^ DW) | | | | | | | | | |
| --- | --- | --- | --- | --- | --- | --- | --- | --- | --- |
|  | Before | | | During | | | After | | |
|  | -Se | +Se | Mean | -Se | +Se | Mean | -Se | +Se | Mean |
| *C. arabica* | 325.3ns | 315.1ns | 320.2a | 287.7ns | 281.8ns | 289.8A | 249.2Aa | 246.7Aa | 247.9A |
| *C. canephora* | 205.8ns | 152.5ns | 179.2b | 145.1ns | 121.3ns | 133.2B | 85.5Bb | 110.31Ab | 97.9B |
| Mean | 265.5ns | 233.8ns |  | 221.4ns | 201.6ns |  | 167.3ns | 178.5ns |  |
| RS (µmol of glucose g^-1^ DW) | | | | | | | | | |
| *C. arabica* | 490.1ns | 483.83ns | 487.0B | 441.1ns | 393.8ns | 417.4ns | 478.9Aa | 562.66Aa | 520.8ns |
| *C. canephora* | 693.82ns | 648.22ns | 671.5A | 385.3ns | 381.1ns | 383.2ns | 359.8Aa | 389.0Ba | 374.5ns |
| Mean | 592.0ns | 566.5ns |  | 413.2ns | 387.5ns |  | 419.3ns | 475.9ns |  |
| TSS (µmol of glucose g^-1^ DW) | | | | | | | | | |
| *C. arabica* | 683.8ns | 619.5ns | 651.7A | 644.7ns | 602.77ns | 623.7A | 608.2ns | 764.5ns | 686.4A |
| *C. canephora* | 457.3ns | 323.8ns | 390.5B | 324.5ns | 266.1ns | 295.3B | 273.8ns | 420.1ns | 346.9B |
| Mean | 570.5a | 471.7a |  | 484.6ns | 434.4ns |  | 441.0b | 592.3a |  |
| Starch (µmol of glucose g^-1^ DW) | | | | | | | | | |
| *C. arabica* | 266.2ns | 234.8.5ns | 250.5A | 293.5ns | 257.5ns | 275.5A | 182.8Ab | 247.6Aa | 215.2A |
| *C. canephora* | 160.5ns | 120.8ns | 140.6B | 136.4s | 119.7ns | 128.1B | 122.4Bb | 151.4Ba | 136.9B |
| Mean | 213.4a | 177.8b |  | 214.9a | 188.6b |  | 152.6b | 199.5a |  |
| Protein (µg protein g^-1^ DW) | | | | | | | | | |
| *C. arabica* | 26.4Bb | 31.05Ba | 28.7B | 21.5ns | 23.0ns | 22.3B | 30.6ns | 26.8ns | 28.7B |
| *C. canephora* | 44.2Aa | 42.2Aa | 43.2A | 37.5ns | 36.3ns | 36.9A | 34.7ns | 33.3ns | 34.0A |
| Mean | 35.3ns | 36.6ns |  | 29.5ns | 29.6ns |  | 32.7ns | 30.1ns |  |
| Proline (µmol proline g^-1^ DW) | | | | | | | | | |
| *C. arabica* | 4.0ns | 3.9ns | 4.0B | 4.5ns | 3.5ns | 4.0B | 9.4Ab | 11.3Aa | 10.3A |
| *C. canephora* | 13.5ns | 13.2ns | 13.3A | 6.9ns | 8.0ns | 7.5A | 3.1Bb | 7.3Ba | 5.2B |
| Mean | 8.8ns | 8.6ns |  | 5.7ns | 5.8ns |  | 6.3b | 9.3a |  |

Mean values followed by different lowercase letters within Se supply conditions (-Se or +Se) in each genotype are significantly different (*p<*0.05, n=5) by Tukey multiple comparison test as well as different uppercase letters that indicate significant differences in genotypes (*C. arabica* and *C. canephora* ). ns = non-significance by Tukey multiple comparison test (*p<*0.05, n=5).

Supplementary Figure 1. Correlation matrix showing Pearson’s correlation of physiological, biochemical, and nutritional parameters of *Coffea arabica* seedlings before the cold shock. Se-Selenium content; S-sulfur content; N-nitrogen content; RS-reducing sugars; AA-total amino acids; Pro-proline; TSS-total soluble sugars; Sta-starch; Suc-sucrose; Prt-protein; APX-ascorbate peroxidase; CAT-catalase; GR-glutathione reductase; SOD-superoxide dismutase; MDA-lipidic peroxidation; HP- hydrogen peroxide.


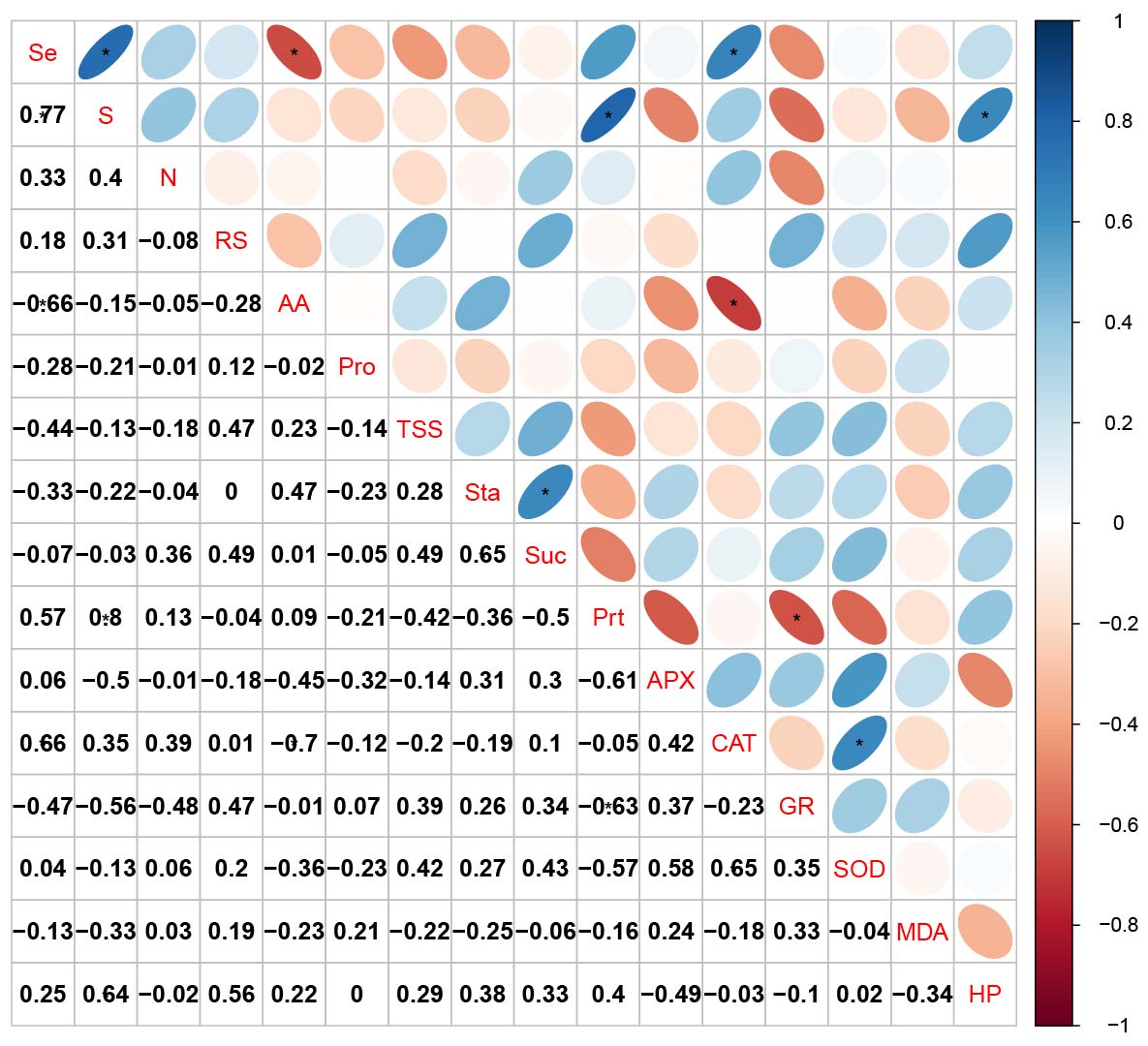


Supplementary Figure 2. Correlation matrix showing Pearson’s correlation of physiological, biochemical, and nutritional parameters of *Coffea canephora s*eedlings before the cold shock. Se-Selenium content; S-sulfur content; N-nitrogen content; RS-reducing sugars; AA-total amino acids; Pro-proline; TSS-total soluble sugars; Sta-starch; Suc-sucrose; Prt-protein; APX-ascorbate peroxidase; CAT-catalase; GR-glutathione reductase; SOD-superoxide dismutase; MDA-lipidic peroxidation; HP- hydrogen peroxide.


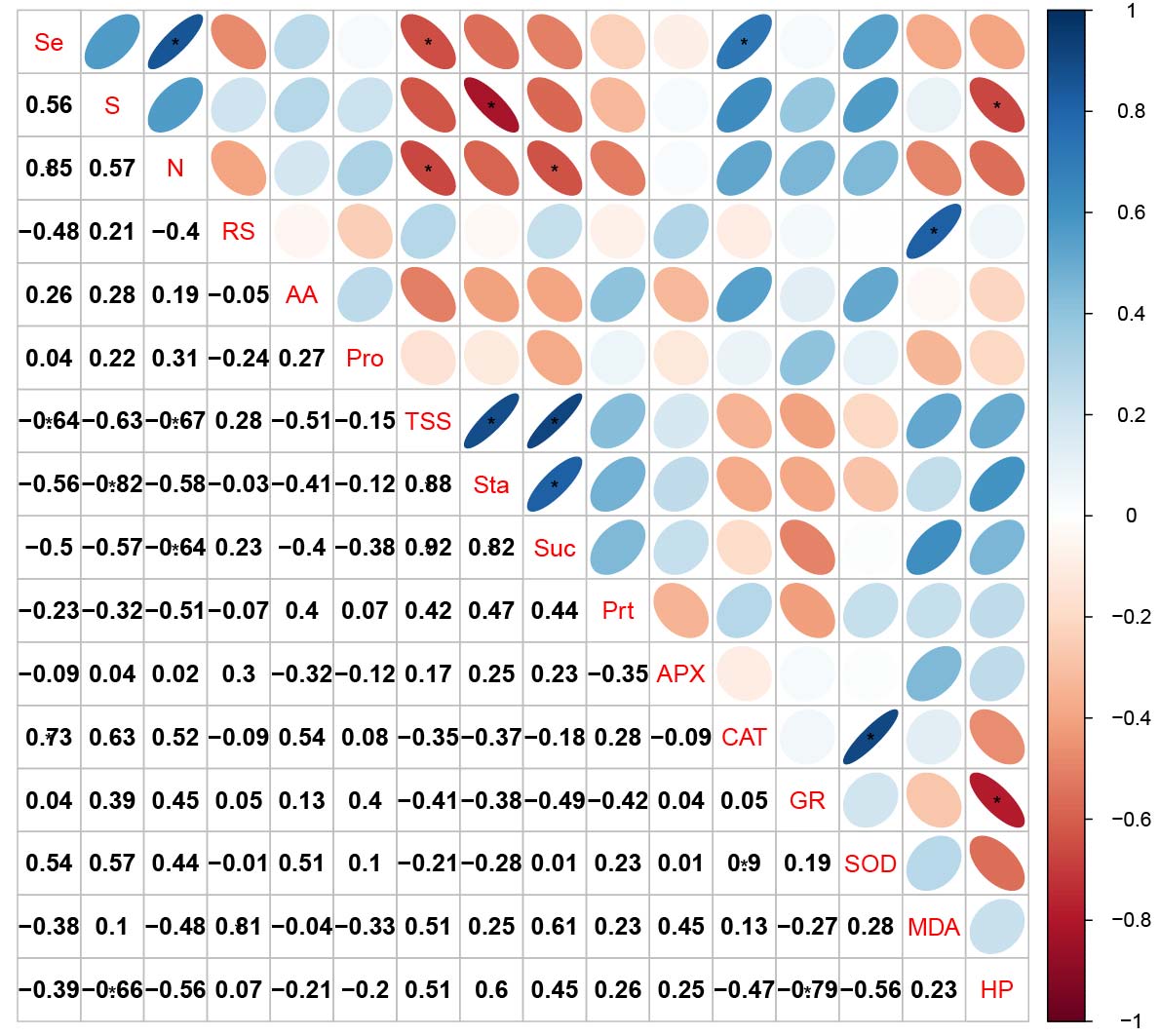


Supplementary Figure 3. Correlation matrix showing Pearson’s correlation of physiological, biochemical, and nutritional parameters of *Coffea arabica s*eedlings during the cold shock. DS=damage scale; Se-Selenium content; S-sulfur content; N-nitrogen content; RS-reducing sugars; AA-total amino acids; Pro-proline; TSS-total soluble sugars; Sta-starch; Suc-sucrose; Prt-protein; APX-ascorbate peroxidase; CAT-catalase; GR-glutathione reductase; SOD-superoxide dismutase; MDA-lipidic peroxidation; HP- hydrogen peroxide.


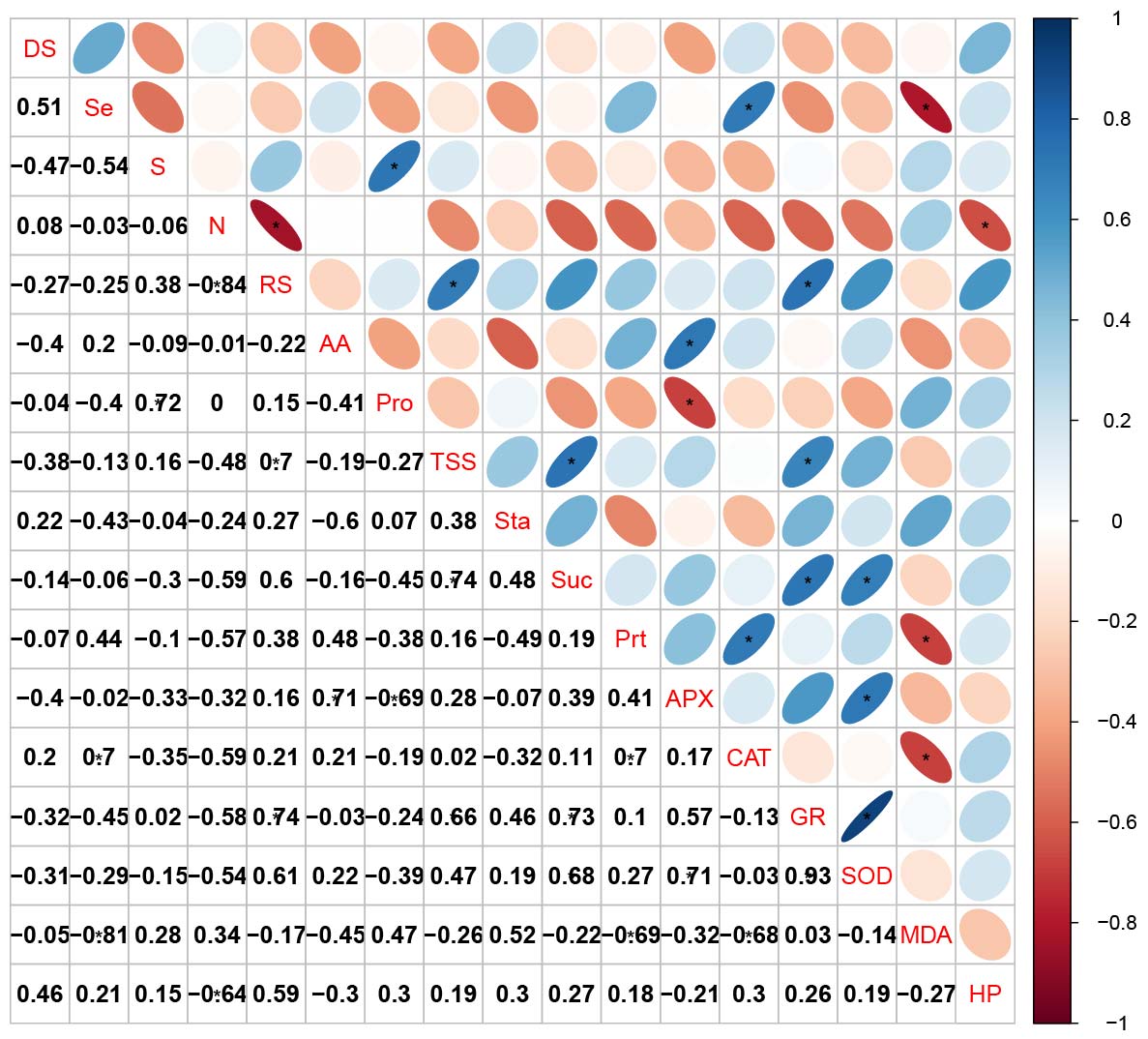


Supplementary Figure 4. Correlation matrix showing Pearson’s correlation of physiological, biochemical, and nutritional parameters of *Coffea canephora s*eedlings during the cold shock. DS=damage scale; Se-Selenium content; S-sulfur content; N-nitrogen content; RS-reducing sugars; AA-total amino acids; Pro-proline; TSS-total soluble sugars; Sta-starch; Suc-sucrose; Prt-protein; APX-ascorbate peroxidase; CAT-catalase; GR-glutathione reductase; SOD-superoxide dismutase; MDA-lipidic peroxidation; HP- hydrogen peroxide.


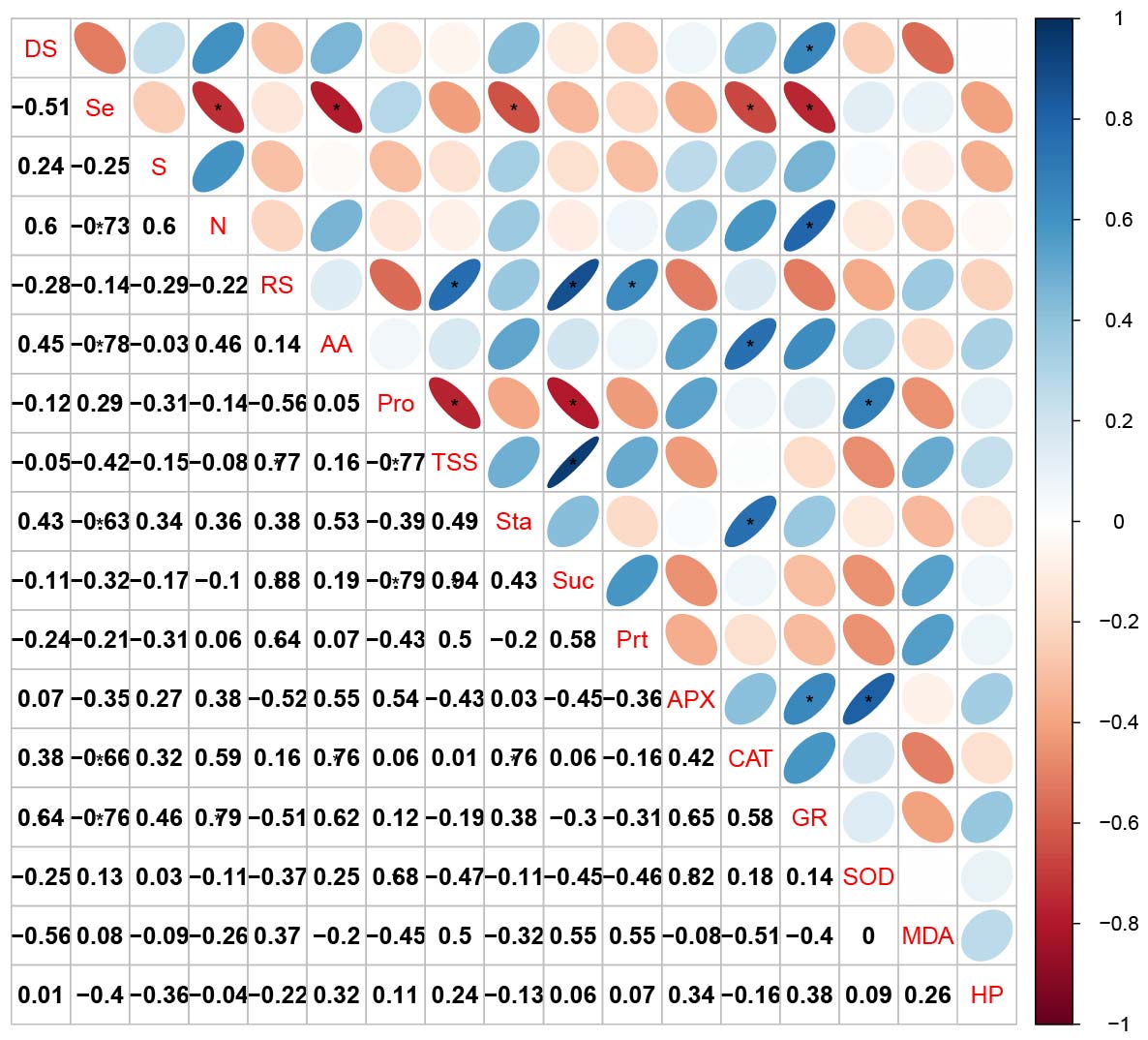


Supplementary Figure 5. Correlation matrix showing Pearson’s correlation of physiological, biochemical, and nutritional parameters of *Coffea arabica s*eedlings after the cold shock. DS=damage scale; Se-Selenium content; S-sulfur content; N-nitrogen content; RS-reducing sugars; AA-total amino acids; Pro-proline; TSS-total soluble sugars; Sta-starch; Suc-sucrose; Prt-protein; APX-ascorbate peroxidase; CAT-catalase; GR-glutathione reductase; SOD-superoxide dismutase; MDA-lipidic peroxidation; HP- hydrogen peroxide.


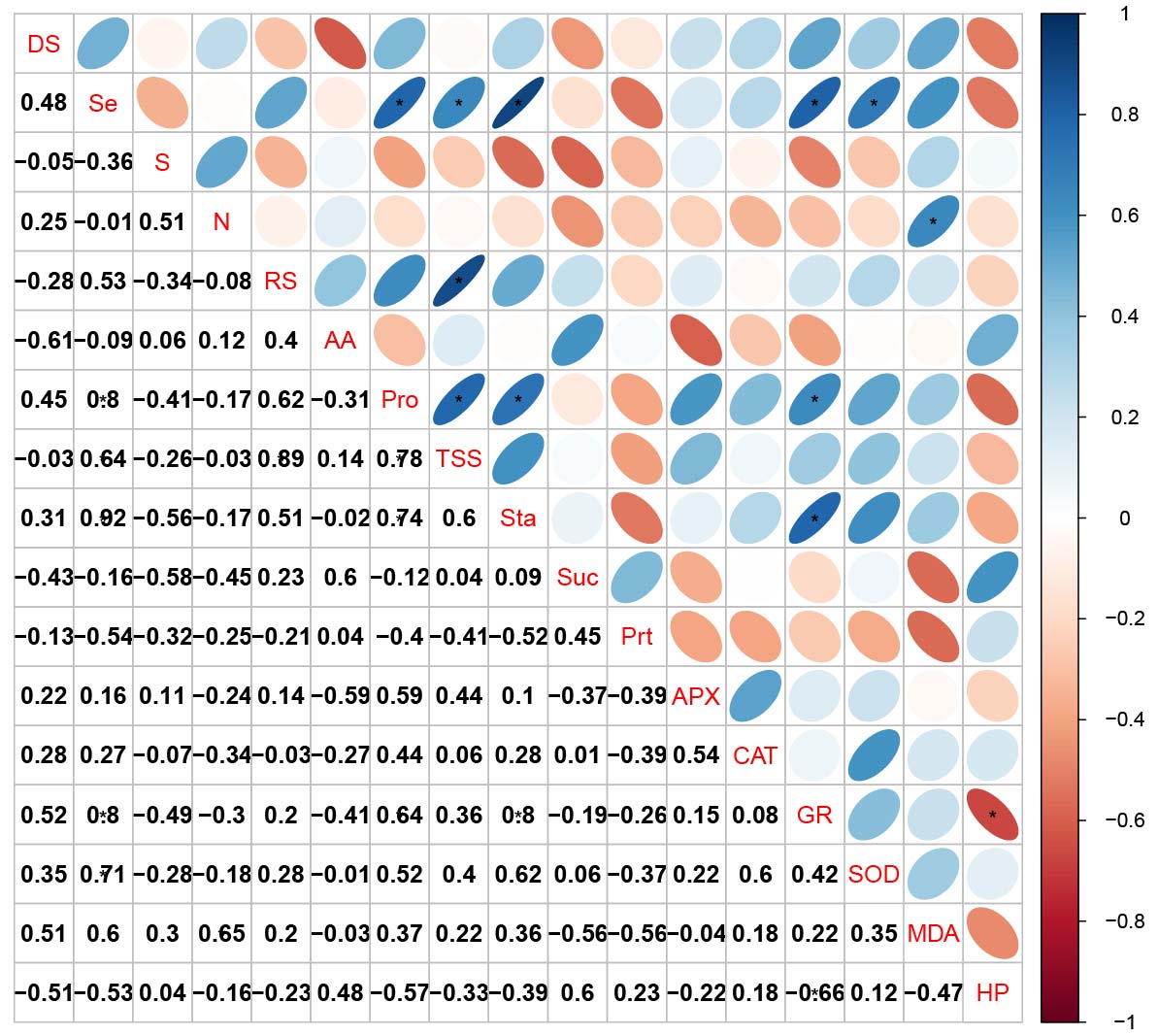


Supplementary Figure 6. Correlation matrix showing Pearson’s correlation of physiological, biochemical, and nutritional parameters of *Coffea canephora s*eedlings after the cold shock. DS=damage scale; Se-Selenium content; S-sulfur content; N-nitrogen content; RS-reducing sugars; AA-total amino acids; Pro-proline; TSS-total soluble sugars; Sta-starch; Suc-sucrose; Prt-protein; APX-ascorbate peroxidase; CAT-catalase; GR-glutathione reductase; SOD-superoxide dismutase; MDA-lipidic peroxidation; HP- hydrogen peroxide.


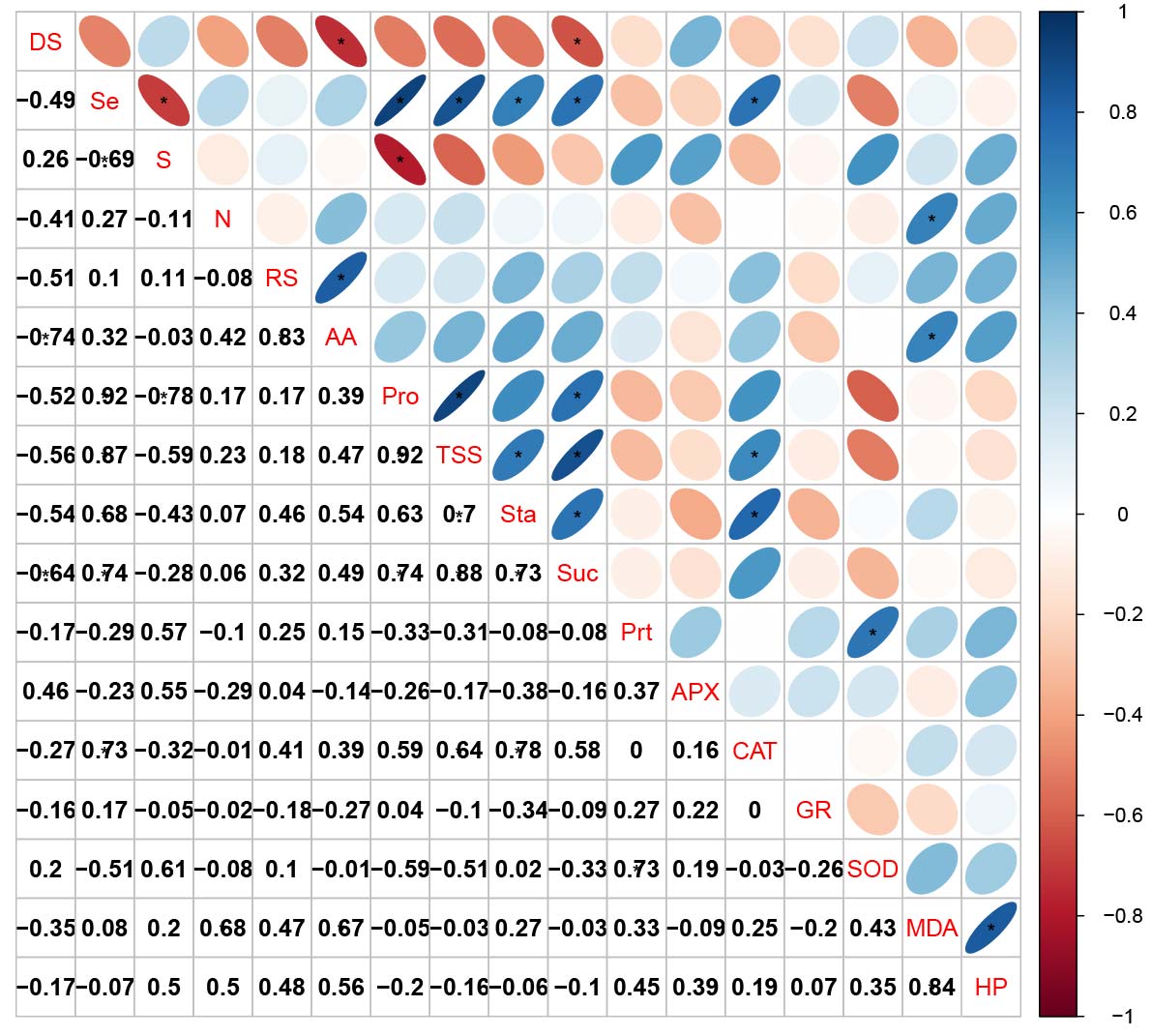

Supplement: Supplementary file 1 [file Data_Sheet_1.docx]
